# Supplementary material for: Extreme temperatures compromise male and female fertility in a large desert bird
Source: Nat Commun. 2021 Feb 2;12:666. doi: 10.1038/s41467-021-20937-7 (PMC7854745; doi:10.1038/s41467-021-20937-7)
Supplement: Supplementary file 1 — Supplementary Information [file 41467_2021_20937_MOESM1_ESM.pdf]

Supplementary Information

Extreme temperatures compromise male and female fertility in a large desert bird

Contents

1 Supplementary Tables **2**

1.1 Supplementary Table 1: Sample sizes within years 2

1.2 Results from random regression models (Supplementary Tables 2-7) 3

1.3 Results from random regression models with year specific slopes (Supplementary Tables 8-11) 9

1.4 Results from character state models (Supplementary Tables 12-17) 13

1.5 Results from two-trait models (Supplementary Tables 18-22) 19

2 Supplementary Figures **26**

2.1 Supplementary Fig. 1: Identification of critical thermal window for egg traits 26

2.2 Supplementary Fig. 2: Identification of critical thermal window for sperm traits 27

2.3 Supplementary Fig. 3: The relationship between ambient temperature and egg-laying. 28

2.4 Supplementary Fig. 4: The relationship between ambient temperature and number of sperm. 29

2.5 Supplementary Fig. 5: Acclimation duration in the start of a new breeding season. 30

2.6 Supplementary Fig. 6: Female age influences egg-laying. 31

2.7 Supplementary Fig. 7: Inspecting ranking of one-day and two-day thermal windows in egg-laying 32

2.8 Supplementary Fig. 8: Change in egg mass with number of days since previous egg. 33

1 Supplementary Tables

1.1 Supplementary Table 1: Sample sizes within years

Yearly number of females and males and observations included in the analysis. Data of egg laying, egg mass, hatchting success and number of offspring were all obtained from the same females every year.

Year	Eggs	Egg mass records	Egg hathcing records	Females	Males (number of sperm)	Males (sperm viability)	Ejaculates (number of sperm)	Ejaculates (sperm viability)
1998	3705	3603	3303	86	0	0	0	0
1999	3506	3421	3310	90	0	0	0	0
2000	2446	2424	2378	48	0	0	0	0
2001	2972	2938	2903	53	0	0	0	0
2002	2221	2193	2123	56	0	0	0	0
2003	4530	4471	4355	102	0	0	0	0
2004	6160	6104	5988	148	0	0	0	0
2005	4702	4641	4369	131	0	0	0	0
2006	5781	5705	5462	124	0	0	0	0
2007	4570	4485	4225	95	0	0	0	0
2008	5470	5366	5024	116	4	4	37	37
2009	4444	4376	4018	100	8	8	358	352
2010	5116	5051	4928	107	7	7	379	380
2011	4147	4107	3981	90	8	7	297	232
2012	2772	2749	2642	71	10	0	79	0
2013	3595	3544	3463	82	8	5	39	16
2014	2974	2920	2880	69	8	0	209	0
2015	2860	2806	2736	65	7	0	20	0
2016	2857	2806	2681	63	10	10	245	225
2017	3164	3116	3074	64	10	8	122	58
2018	3547	3512	3463	70	0	0	0	0

1.2 Results from random regression models (Supplementary Tables 2-7)

1.2.1 Supplementary Table 2: Egg laying (random regression)

Testing the effect of thermal stress on egg laying rate using a random slope model in the R-package MCMCglmm.

Type	Parameter	Posterior mode (CI)	pMCMC	Level
**Fixed effects**	Intercept	0.26 (0.08,0.42)	**0.006**	-
	dam_subpopZB	-0.39 (-0.67,-0.18)	**0.001**	-
	dam_subpopCross	-0.15 (-0.36,0.04)	0.141	-
	sire_subpopZB	-0.13 (-0.24,0)	0.051	-
	sire_subpopCross	-0.09 (-0.29,0.12)	0.4	-
	dam_age_z	-0.04 (-0.07,0)	**0.034**	-
	TSIcon:StressCold	-1.97 (-2.33,-1.61)	**0.001**	-
	TSIcon:StressHeat	-1.62 (-1.81,-1.41)	**0.001**	-
	TSIcon:StressCold:dam_subpopZB	-0.37 (-1.57,0.64)	0.507	-
	TSIcon:StressHeat:dam_subpopZB	-0.37 (-0.96,0.26)	0.246	-
	TSIcon:StressCold:dam_subpopCross	-1.36 (-2.28,-0.45)	**0.004**	-
	TSIcon:StressHeat:dam_subpopCross	0.38 (-0.15,0.91)	0.164	-
**Random effect var.**	(Intercept)	0.564 (0.511,0.694)	-	damid
	TSIcon:StressCold	2.139 (1.247,3.32)	-	damid
	TSIcon:StressHeat	2.956 (2.32,3.53)	-	damid
	enclosure	0.179 (0.14,0.238)	-	enclosure
	year	0.092 (0.037,0.156)	-	year
	residuals	0.603 (0.579,0.641)	-	units
**Correlations**	TSIcon:StressCold:(Intercept).damid	0.136 (-0.08,0.361)	0.172	-
	TSIcon:StressHeat:(Intercept).damid	-0.072 (-0.208,0.044)	0.154	-
**Fixed effect var.**	All fixed - thermal stress	0.027 (0.013,0.06)	-	-
**Expected scale**	Intercept individual variance	4.967 (4.505,5.822)	-	-
	Intercept phenotypic variance	10.18 (9.519,10.717)	-	-
	Intercept repeatability	0.519 (0.463,0.556)	-	-

For fixed effects the posterior mean is reported

1.2.2 Supplementary Table 3: Number of sperm (random regression)

Testing the effect of thermal stress on number of sperm using a random slope model in the R-package MCMCglmm.

Type	Parameter	Posterior mode (CI)	pMCMC	Level
**Fixed effects**	Intercept	10.47 (10.17,10.84)	**0.001**	-
	poly(age, 2)1	1.62 (-2.56,6.24)	0.503	-
	poly(age, 2)2	-5.47 (-8.18,-3.22)	**0.001**	-
	TSI:StressCold	-0.63 (-1.07,-0.24)	**0.002**	-
	TSI:StressHeat	-0.67 (-1.24,-0.02)	**0.028**	-
**Random effect var.**	(Intercept)	0.265 (0.157,0.663)	-	name
	TSI:StressCold	0.24 (0.123,0.871)	-	name
	TSI:StressHeat	0.705 (0.231,2.878)	-	name
	year	0.049 (0.015,0.197)	-	year
	residuals	0.923 (0.86,0.987)	-	units
**Correlations**	TSI:StressCold:(Intercept).name	-0.162 (-0.583,0.49)	0.786	-
	TSI:StressHeat:(Intercept).name	-0.111 (-0.571,0.468)	0.921	-
**Fixed effect var.**	All fixed - thermal stress	0.018 (0.004,0.041)	-	-
**Expected scale**	Intercept individual variance	487.713 (166.046,2733.395)	-	-

For fixed effects the posterior mean is reported

1.2.3 Supplementary Table 4: Egg mass (random regression)

Testing the effect of thermal stress on egg mass using a random slope model in the R-package MCMCglmm.

Type	Parameter	Posterior mode (CI)	pMCMC	Level
**Fixed effects**	Intercept	1.43 (1.42,1.45)	**0.001**	-
	dam__subpopZB	0.07 (0.04,0.11)	**0.001**	-
	dam__subpopCross	0.04 (0.01,0.07)	**0.001**	-
	sire__subpopZB	0.01 (0.01,0.02)	**0.001**	-
	sire__subpopCross	0 (0,0.01)	0.404	-
	poly(days.since.prev.eggLOG__z, 2)1	-5.15 (-5.3,-5)	**0.001**	-
	poly(days.since.prev.eggLOG__z, 2)2	-1.85 (-1.98,-1.7)	**0.001**	-
	dam__age__z	0 (-0.01,0)	**0.004**	-
	TSI:StressCold	-0.05 (-0.06,-0.03)	**0.001**	-
	TSI:StressHeat	0.01 (0,0.02)	0.261	-
	TSI:StressCold:dam__subpopZB	0.02 (-0.04,0.07)	0.461	-
	TSI:StressHeat:dam__subpopZB	-0.02 (-0.05,0.01)	0.301	-
	TSI:StressCold:dam__subpopCross	-0.02 (-0.06,0.02)	0.307	-
	TSI:StressHeat:dam__subpopCross	0.01 (-0.02,0.03)	0.497	-
**Random effect var.**	(Intercept)	0.017 (0.015,0.019)	-	damid
	TSI:StressCold	0.021 (0.018,0.025)	-	damid
	TSI:StressHeat	0.012 (0.01,0.013)	-	damid
	enclosure	0.002 (0.002,0.003)	-	enclosure
	year	0 (0,0.001)	-	year
	residuals	0.005 (0.005,0.005)	-	units
**Correlations**	TSI:StressCold:(Intercept).damid	-0.082 (-0.2,-0.012)	**0.032**	-
	TSI:StressHeat:(Intercept).damid	-0.024 (-0.112,0.053)	0.463	-
**Fixed effect var.**	All fixed - thermal stress	0.001 (0.001,0.002)	-	-

For fixed effects the posterior mean is reported

1.2.4 Supplementary Table 5: Sperm viability (random regression)

Testing the effect of thermal stress on sperm viability using a random slope model in the R-package MCMCglmm.

Type	Parameter	Posterior mode (CI)	pMCMC	Level
**Fixed effects**	Intercept	-1.91 (-2.23,-1.61)	**0.001**	-
	poly(age, 2)1	-2.64 (-5.45,-0.16)	**0.044**	-
	poly(age, 2)2	1.46 (0.16,2.57)	**0.014**	-
	TSI:StressCold	-0.24 (-0.55,0.08)	0.152	-
	TSI:StressHeat	0.06 (-0.22,0.29)	0.651	-
**Random effect var.**	(Intercept)	0.165 (0.073,0.352)	-	name
	TSI:StressCold	0.366 (0.134,0.707)	-	name
	TSI:StressHeat	0.18 (0.083,0.441)	-	name
	year	0.032 (0.016,0.252)	-	year
	residuals	0.039 (0.034,0.043)	-	units
**Correlations**	TSI:StressCold:(Intercept).name	0.279 (-0.402,0.571)	0.671	-
	TSI:StressHeat:(Intercept).name	-0.267 (-0.681,0.287)	0.527	-
**Fixed effect var.**	All fixed - thermal stress	0.004 (0,0.021)	-	-
**Expected scale**	Intercept individual variance	631.663 (197.463,1409.6)	-	-

For fixed effects the posterior mean is reported

1.2.5 Supplementary Table 6: Hatching success (random regression)

Testing the effect of thermal stress on hatching success using a random slope model in the R-package MCMCglmm.

Type	Parameter	Posterior mode (CI)	pMCMC	Level
**Fixed effects**	Intercept	-0.04 (-0.25,0.18)	0.685	-
	dam_subpopZB	0.12 (-0.24,0.43)	0.501	-
	dam_subpopCross	0.05 (-0.24,0.32)	0.705	-
	sire_subpopZB	0.16 (-0.01,0.32)	0.069	-
	sire_subpopCross	0.45 (0.17,0.69)	**0.001**	-
	dam_age_z	-0.1 (-0.14,-0.05)	**0.001**	-
	TSIcon:StressCold	-0.57 (-0.98,0.01)	**0.028**	-
	TSIcon:StressHeat	-0.26 (-0.43,-0.09)	**0.002**	-
	TSIcon:StressCold:dam_subpopZB	-0.15 (-1.54,1.47)	0.826	-
	TSIcon:StressHeat:dam_subpopZB	-0.14 (-0.67,0.41)	0.628	-
	TSIcon:StressCold:dam_subpopCross	-1.46 (-2.73,-0.36)	**0.012**	-
	TSIcon:StressHeat:dam_subpopCross	-0.2 (-0.62,0.26)	0.382	-
**Random effect var.**	(Intercept)	1.164 (0.99,1.363)	-	damid
	TSIcon:StressCold	0.784 (0.299,2.943)	-	damid
	TSIcon:StressHeat	0.728 (0.376,0.977)	-	damid
	enclosure	0.341 (0.236,0.424)	-	enclosure
	year	0.134 (0.07,0.282)	-	year
	residuals	0.556 (0.519,0.61)	-	units

For fixed effects the posterior mean is reported

1.2.6 Supplementary Table 7: Number of offspring (random regression)

Testing the effect of thermal stress on number of offspring using a random slope model in the R-package MCMCglmm.

Type	Parameter	Posterior mode (CI)	pMCMC	Level
**Fixed effects**	Intercept	-1.25 (-1.46,-1.05)	**0.001**	-
	dam_subpopZB	-0.18 (-0.47,0.14)	0.253	-
	dam_subpopCross	-0.08 (-0.34,0.18)	0.501	-
	sire_subpopZB	0.04 (-0.11,0.18)	0.636	-
	sire_subpopCross	0.25 (0.01,0.48)	0.051	-
	dam_age_z	-0.07 (-0.12,-0.04)	**0.001**	-
	TSIcon:StressCold	-2.1 (-2.57,-1.6)	**0.001**	-
	TSIcon:StressHeat	-1.42 (-1.61,-1.21)	**0.001**	-
	TSIcon:StressCold:dam_subpopZB	-0.66 (-2.12,0.67)	0.341	-
	TSIcon:StressHeat:dam_subpopZB	-0.51 (-1.08,0.07)	0.087	-
	TSIcon:StressCold:dam_subpopCross	-1.88 (-3.01,-0.78)	**0.008**	-
	TSIcon:StressHeat:dam_subpopCross	0.04 (-0.43,0.48)	0.885	-
**Random effect var.**	(Intercept)	1.058 (0.884,1.23)	-	damid
	TSIcon:StressCold	2.419 (1.14,4.239)	-	damid
	TSIcon:StressHeat	1.889 (1.458,2.443)	-	damid
	enclosure	0.3 (0.224,0.383)	-	enclosure
	year	0.106 (0.047,0.212)	-	year
	residuals	0.703 (0.663,0.747)	-	units

For fixed effects the posterior mean is reported

1.3 Results from random regression models with year specific slopes (Supplementary Tables 8-11)

1.3.1 Supplementary Table 8: Egg laying (year specific random regression)

Testing the effect of thermal stress on egg laying rate using a (yearly) random slope model in the R-package MCMCglmm.

Type	Parameter	Posterior mode (CI)	pMCMC	Level
**Fixed effects**	Intercept	0.32 (0.18,0.49)	**0.001**	-
	dam_subpopZB	-0.39 (-0.6,-0.18)	**0.001**	-
	dam_subpopCross	-0.16 (-0.33,0.04)	0.091	-
	sire_subpopZB	-0.17 (-0.34,0)	**0.046**	-
	sire_subpopCross	-0.23 (-0.49,0.04)	0.083	-
	dam_age_z	0.01 (-0.04,0.05)	0.756	-
	TSIcon:StressCold	-2.1 (-2.43,-1.8)	**0.001**	-
	TSIcon:StressHeat	-1.68 (-1.9,-1.5)	**0.001**	-
	TSIcon:StressCold:dam_subpopZB	-0.39 (-1.24,0.62)	0.402	-
	TSIcon:StressHeat:dam_subpopZB	-0.45 (-1.01,0.16)	0.143	-
	TSIcon:StressCold:dam_subpopCross	-1.28 (-2.03,-0.58)	**0.001**	-
	TSIcon:StressHeat:dam_subpopCross	0.42 (-0.06,0.91)	0.091	-
**Random effect var.**	(Intercept)	0.311 (0.244,0.384)	-	damid
	TSIcon:StressCold	1.345 (0.457,2.22)	-	damid
	TSIcon:StressHeat	1.609 (1.146,2.275)	-	damid
	(Intercept)	0.484 (0.427,0.548)	-	year_damid
	TSIcon:StressCold	4.339 (3.103,7.009)	-	year_damid
	TSIcon:StressHeat	4.963 (4.411,5.794)	-	year_damid
	enclosure	0.087 (0.054,0.134)	-	enclosure
	year	0.073 (0.034,0.157)	-	year
	residuals	0.163 (0.147,0.184)	-	units
**Repeatabilities**	Intercept	0.275 (0.216,0.324)	-	-
	Heat stress slope	0.244 (0.174,0.322)	-	-
	Cold stress slope	0.177 (0.06,0.341)	-	-
**Fixed effect var.**	All fixed - thermal stress	0.036 (0.013,0.062)	-	-
**Expected scale**	Intercept individual variance	2.919 (2.377,3.57)	-	-
	Intercept phenotypic variance	8.463 (7.88,9.2)	-	-
	Intercept repeatability	0.354 (0.284,0.409)	-	-

For fixed effects the posterior mean is reported

1.3.2 Supplementary Table 9: Number of sperm (year specific random regression)

Testing the effect of thermal stress on number of sperm using a (yearly) random slope model in the R-package MCMCglmm.

Type	Parameter	Posterior mode (CI)	pMCMC	Level
**Fixed effects**	Intercept	10.45 (10.11,10.76)	**0.001**	-
	poly(age, 2)1	2.52 (-2.46,8.23)	0.374	-
	poly(age, 2)2	-6.2 (-10.37,-2.03)	**0.006**	-
	TSI:StressCold	-0.67 (-1.18,-0.23)	**0.008**	-
	TSI:StressHeat	-0.67 (-1.32,-0.07)	**0.032**	-
**Random effect var.**	(Intercept)	0.309 (0.131,0.658)	-	name
	TSI:StressCold	0.299 (0.131,0.982)	-	name
	TSI:StressHeat	0.762 (0.188,2.469)	-	name
	(Intercept)	0.16 (0.088,0.258)	-	year_name
	TSI:StressCold	0.286 (0.134,0.851)	-	year_name
	TSI:StressHeat	0.771 (0.18,2.15)	-	year_name
	year	0.002 (0,0.114)	-	year
	residuals	0.883 (0.812,0.933)	-	units
**Repeatabilities**	Intercept	0.232 (0.137,0.404)	-	-
	Heat stress slope	0.493 (0.136,0.831)	-	-
	Cold stress slope	0.414 (0.254,0.793)	-	-
**Fixed effect var.**	All fixed - thermal stress	0.011 (0.002,0.067)	-	-
**Expected scale**	Intercept individual variance	544.614 (143.844,2543.709)	-	-
	Intercept phenotypic variance	12684.275 (5969.37,40455.823)	-	-
	Intercept repeatability	0.039 (0.021,0.076)	-	-

For fixed effects the posterior mean is reported

1.3.3 Supplementary Table 10: Egg mass (year specific random regression)

Testing the effect of thermal stress on egg mass using a (yearly) random slope model in the R-package MCMCglmm.

Type	Parameter	Posterior mode (CI)	pMCMC	Level
**Fixed effects**	Intercept	1.43 (1.42,1.45)	**0.001**	-
	dam__subpopZB	0.09 (0.05,0.12)	**0.001**	-
	dam__subpopCross	0.05 (0.02,0.07)	**0.002**	-
	sire__subpopZB	0.02 (0,0.03)	**0.036**	-
	sire__subpopCross	0 (-0.02,0.03)	0.857	-
	poly(days.since.prev.eggLOG__z, 2)1	-5.03 (-5.16,-4.89)	**0.001**	-
	poly(days.since.prev.eggLOG__z, 2)2	-1.86 (-2,-1.72)	**0.001**	-
	dam__age__z	-0.01 (-0.01,0)	**0.02**	-
	TSI:StressCold	-0.05 (-0.07,-0.03)	**0.001**	-
	TSI:StressHeat	0 (-0.01,0.02)	0.572	-
	TSI:StressCold:dam__subpopZB	0.01 (-0.04,0.07)	0.675	-
	TSI:StressHeat:dam__subpopZB	-0.03 (-0.06,0.01)	0.152	-
	TSI:StressCold:dam__subpopCross	-0.02 (-0.07,0.02)	0.275	-
	TSI:StressHeat:dam__subpopCross	0.01 (-0.01,0.04)	0.378	-
**Random effect var.**	(Intercept)	0.015 (0.013,0.017)	-	damid
	TSI:StressCold	0.019 (0.015,0.022)	-	damid
	TSI:StressHeat	0.011 (0.009,0.012)	-	damid
	(Intercept)	0.004 (0.004,0.004)	-	year__damid
	TSI:StressCold	0.021 (0.018,0.024)	-	year__damid
	TSI:StressHeat	0.01 (0.009,0.011)	-	year__damid
	enclosure	0 (0,0)	-	enclosure
	year	0 (0,0.001)	-	year
	residuals	0.004 (0.004,0.005)	-	units
**Repeatabilities**	Intercept	0.622 (0.588,0.652)	-	-
	Heat stress slope	0.525 (0.482,0.57)	-	-
	Cold stress slope	0.474 (0.41,0.528)	-	-
**Fixed effect var.**	All fixed - thermal stress	0.001 (0.001,0.002)	-	-

For fixed effects the posterior mean is reported

1.3.4 Supplementary Table 11: Sperm viability (year specific random regression)

Testing the effect of thermal stress on sperm viability using a (yearly) random slope model in the R-package MCMCglmm.

Type	Parameter	Posterior mode (CI)	pMCMC	Level
**Fixed effects**	Intercept	-1.93 (-2.24,-1.67)	**0.001**	-
	poly(age, 2)1	-3.53 (-7.92,0.29)	0.099	-
	poly(age, 2)2	0.25 (-4.05,4.3)	0.909	-
	TSI:StressCold	-0.2 (-0.53,0.11)	0.242	-
	TSI:StressHeat	0.13 (-0.18,0.48)	0.392	-
**Random effect var.**	(Intercept)	0.155 (0.084,0.381)	-	name
	TSI:StressCold	0.293 (0.096,0.625)	-	name
	TSI:StressHeat	0.208 (0.1,0.581)	-	name
	(Intercept)	0.094 (0.06,0.179)	-	year_name
	TSI:StressCold	0.153 (0.091,0.341)	-	year_name
	TSI:StressHeat	0.154 (0.085,0.341)	-	year_name
	year	0.004 (0,0.159)	-	year
	residuals	0.036 (0.03,0.039)	-	units
**Repeatabilities**	Intercept	0.592 (0.31,0.727)	-	-
	Heat stress slope	0.652 (0.359,0.82)	-	-
	Cold stress slope	0.615 (0.364,0.815)	-	-
**Fixed effect var.**	All fixed - thermal stress	0.001 (0,0.04)	-	-
**Expected scale**	Intercept individual variance	466.637 (229.317,1463.239)	-	-
	Intercept phenotypic variance	1246.648 (718.613,2649.615)	-	-
	Intercept repeatability	0.532 (0.277,0.702)	-	-

For fixed effects the posterior mean is reported

1.4 Results from character state models (Supplementary Tables 12-17)

1.4.1 Supplementary Table 12: Egg laying (character-state)

Testing the effect of thermal stress on egg laying rate using a character-state model in the R-package MCMCglmm.

Type	Parameter	Posterior mode (CI)	pMCMC	Level
**Fixed effects**	Intercept	0.08 (-0.07,0.25)	0.313	-
	TSIcatCold	-0.05 (-0.14,0.03)	0.251	-
	TSIcatHot	-0.64 (-0.72,-0.56)	**0.001**	-
	dam_subpopZB	-0.45 (-0.66,-0.2)	**0.001**	-
	dam_subpopCross	-0.13 (-0.32,0.05)	0.192	-
	sire_subpopZB	-0.14 (-0.29,0)	0.055	-
	sire_subpopCross	-0.15 (-0.38,0.08)	0.212	-
	dam_age_z	-0.03 (-0.07,0.01)	0.22	-
	TSIcatCold:dam_subpopZB	-0.07 (-0.32,0.2)	0.582	-
	TSIcatHot:dam_subpopZB	-0.17 (-0.41,0.08)	0.188	-
	TSIcatCold:dam_subpopCross	-0.26 (-0.46,-0.06)	**0.016**	-
	TSIcatHot:dam_subpopCross	0.14 (-0.06,0.36)	0.204	-
**Random effect var.**	TSIcatBenign	0.505 (0.43,0.585)	-	damid
	TSIcatCold	0.541 (0.441,0.711)	-	damid
	TSIcatHot	0.884 (0.694,1.03)	-	damid
	enclosure	0.142 (0.098,0.189)	-	enclosure
	year	0.08 (0.041,0.18)	-	year
	TSIcatBenign	0.363 (0.335,0.399)	-	units
	TSIcatCold	0.93 (0.821,1.043)	-	units
	TSIcatHot	0.857 (0.789,0.982)	-	units
**Correlations**	TSIcatCold:TSIcatBenign.damid	0.849 (0.783,0.883)	**0.001**	-
	TSIcatHot:TSIcatBenign.damid	0.877 (0.834,0.915)	**0.001**	-

For fixed effects the posterior mean is reported

1.4.2 Supplementary Table 13: Number of sperm (character-state)

Testing the effect of thermal stress on number of sperm using a character-state model in the R-package MCMCglmm.

Type	Parameter	Posterior mode (CI)	pMCMC	Level
**Fixed effects**	Intercept	10.39 (10.06,10.74)	**0.001**	-
	TSIcatCold	-0.35 (-0.66,0)	**0.034**	-
	TSIcatHot	-0.19 (-0.55,0.16)	0.253	-
	poly(age, 2)1	2.56 (-2.08,6.98)	0.279	-
	poly(age, 2)2	-5.11 (-7.53,-2.38)	**0.001**	-
**Random effect var.**	TSIcatBenign	0.304 (0.171,0.645)	-	name
	TSIcatCold	0.396 (0.172,0.811)	-	name
	TSIcatHot	0.417 (0.224,1.064)	-	name
	year	0.074 (0.015,0.246)	-	year
	TSIcatBenign	0.827 (0.763,0.901)	-	units
	TSIcatCold	0.892 (0.778,1.096)	-	units
	TSIcatHot	1.232 (1.09,1.471)	-	units
**Correlations**	TSIcatCold:TSIcatBenign.name	0.587 (0.127,0.824)	**0.026**	-
	TSIcatHot:TSIcatBenign.name	0.563 (0.061,0.786)	**0.046**	-

For fixed effects the posterior mean is reported

1.4.3 Supplementary Table 14: Egg mass (character-state)

Testing the effect of thermal stress on egg mass using a character-state model in the R-package MCMCglmm.

Type	Parameter	Posterior mode (CI)	pMCMC	Level
**Fixed effects**	Intercept	1.44 (1.42,1.45)	**0.001**	-
	TSIcatCold	-0.01 (-0.02,0)	**0.034**	-
	TSIcatHot	0 (-0.01,0.01)	0.646	-
	dam__subpopZB	0.07 (0.04,0.1)	**0.001**	-
	dam__subpopCross	0.04 (0.01,0.07)	**0.006**	-
	sire__subpopZB	0.01 (0.01,0.02)	**0.001**	-
	sire__subpopCross	0 (0,0.01)	0.384	-
	poly(days.since.prev.eggLOG__z, 2)1	-5.17 (-5.32,-5.02)	**0.001**	-
	poly(days.since.prev.eggLOG__z, 2)2	-1.85 (-1.99,-1.68)	**0.001**	-
	dam__age__z	0 (-0.01,0)	**0.001**	-
	TSIcatCold:dam__subpopZB	0.01 (-0.02,0.03)	0.642	-
	TSIcatHot:dam__subpopZB	0 (-0.03,0.02)	0.749	-
	TSIcatCold:dam__subpopCross	-0.01 (-0.03,0.01)	0.57	-
	TSIcatHot:dam__subpopCross	0 (-0.02,0.02)	0.8	-
**Random effect var.**	TSIcatBenign	0.017 (0.015,0.019)	-	damid
	TSIcatCold	0.017 (0.015,0.019)	-	damid
	TSIcatHot	0.019 (0.016,0.021)	-	damid
	enclosure	0.002 (0.002,0.002)	-	enclosure
	year	0 (0,0.001)	-	year
	TSIcatBenign	0.005 (0.005,0.005)	-	units
	TSIcatCold	0.006 (0.006,0.006)	-	units
	TSIcatHot	0.005 (0.005,0.006)	-	units
**Correlations**	TSIcatCold:TSIcatBenign.damid	0.761 (0.733,0.795)	**0.001**	-
	TSIcatHot:TSIcatBenign.damid	0.767 (0.738,0.803)	**0.001**	-

For fixed effects the posterior mean is reported

1.4.4 Supplementary Table 15: Sperm viability (character-state)

Testing the effect of thermal stress on sperm viability using a character-state model in the R-package MCMCglmm.

Type	Parameter	Posterior mode (CI)	pMCMC	Level
**Fixed effects**	Intercept	-1.93 (-2.19,-1.6)	**0.001**	-
	TSIcatCold	-0.11 (-0.41,0.17)	0.455	-
	TSIcatHot	0.03 (-0.23,0.3)	0.842	-
	poly(age, 2)1	-2.3 (-4.48,0.16)	0.061	-
	poly(age, 2)2	1.29 (0.03,2.67)	0.067	-
**Random effect var.**	TSIcatBenign	0.167 (0.092,0.379)	-	name
	TSIcatCold	0.203 (0.106,0.484)	-	name
	TSIcatHot	0.143 (0.074,0.341)	-	name
	year	0.052 (0.014,0.224)	-	year
	TSIcatBenign	0.035 (0.031,0.041)	-	units
	TSIcatCold	0.089 (0.073,0.12)	-	units
	TSIcatHot	0.051 (0.043,0.067)	-	units
**Correlations**	TSIcatCold:TSIcatBenign.name	0.426 (-0.1,0.721)	0.176	-
	TSIcatHot:TSIcatBenign.name	0.215 (-0.265,0.631)	0.418	-

For fixed effects the posterior mean is reported

1.4.5 Supplementary Table 16: Hatching success (character-state)

Testing the effect of thermal stress on hatching success using a character-state model in the R-package MCMCglmm.

Type	Parameter	Posterior mode (CI)	pMCMC	Level
**Fixed effects**	Intercept	-0.11 (-0.31,0.09)	0.226	-
	TSIcatCold	-0.09 (-0.18,0.02)	0.101	-
	TSIcatHot	-0.05 (-0.13,0.05)	0.325	-
	dam_subpopZB	0.05 (-0.24,0.4)	0.743	-
	dam_subpopCross	-0.02 (-0.28,0.24)	0.907	-
	sire_subpopZB	0.22 (0.05,0.41)	**0.016**	-
	sire_subpopCross	0.49 (0.21,0.82)	**0.002**	-
	dam_age_z	-0.09 (-0.14,-0.03)	**0.001**	-
	TSIcatCold:dam_subpopZB	0.02 (-0.31,0.31)	0.867	-
	TSIcatHot:dam_subpopZB	-0.01 (-0.27,0.3)	0.917	-
	TSIcatCold:dam_subpopCross	-0.13 (-0.39,0.1)	0.317	-
	TSIcatHot:dam_subpopCross	-0.02 (-0.27,0.2)	0.927	-
**Random effect var.**	TSIcatBenign	0.906 (0.761,1.078)	-	damid
	TSIcatCold	0.961 (0.798,1.254)	-	damid
	TSIcatHot	1.027 (0.813,1.231)	-	damid
	enclosure	0.192 (0.135,0.275)	-	enclosure
	year	0.091 (0.06,0.237)	-	year
	TSIcatBenign	0.628 (0.57,0.703)	-	units
	TSIcatCold	0.652 (0.527,0.835)	-	units
	TSIcatHot	0.613 (0.49,0.723)	-	units

For fixed effects the posterior mean is reported

1.4.6 Supplementary Table 17: Number of offspring (character-state)

Testing the effect of thermal stress on number of offspring using a character-state model in the R-package MCMCglmm.

Type	Parameter	Posterior mode (CI)	pMCMC	Level
**Fixed effects**	Intercept	-1.41 (-1.59,-1.23)	**0.001**	-
	TSIcatCold	-0.26 (-0.36,-0.16)	**0.001**	-
	TSIcatHot	-0.66 (-0.77,-0.57)	**0.001**	-
	dam_subpopZB	-0.26 (-0.54,0.02)	0.071	-
	dam_subpopCross	-0.12 (-0.35,0.11)	0.341	-
	sire_subpopZB	0.05 (-0.12,0.22)	0.547	-
	sire_subpopCross	0.26 (-0.03,0.52)	0.077	-
	dam_age_z	-0.07 (-0.11,-0.02)	**0.006**	-
	TSIcatCold:dam_subpopZB	-0.01 (-0.28,0.25)	0.956	-
	TSIcatHot:dam_subpopZB	-0.14 (-0.41,0.12)	0.307	-
	TSIcatCold:dam_subpopCross	-0.28 (-0.5,-0.05)	**0.014**	-
	TSIcatHot:dam_subpopCross	0.09 (-0.14,0.33)	0.442	-
**Random effect var.**	TSIcatBenign	0.891 (0.744,1.023)	-	damid
	TSIcatCold	0.995 (0.839,1.29)	-	damid
	TSIcatHot	1.184 (0.983,1.468)	-	damid
	enclosure	0.221 (0.164,0.3)	-	enclosure
	year	0.088 (0.05,0.209)	-	year
	TSIcatBenign	0.643 (0.591,0.699)	-	units
	TSIcatCold	1.021 (0.846,1.166)	-	units
	TSIcatHot	1.039 (0.925,1.198)	-	units

For fixed effects the posterior mean is reported

1.5 Results from two-trait models (Supplementary Tables 18-22)

1.5.1 Supplementary Table 18: Phenotypic correlations from character state two-trait models

Testing for trade-offs between fertility traits in cold and hot environments. Phenotypic correlations among and within individuals were estimated using two-trait character-state models in MCMCglmm. Credible intervals not overlapping with zero are written in bold. See supplementary tables 19-20 below for model details.

**Trait compairon**	**Level**	**Cold**	**Benign**	**Hot**
**Eggs laying vs eggmass**	Among individuals	0.068 (-0.098,0.186)	0.105 (-0.015,0.193)	0.147 (-0.011,0.251)
	Within individuals	**0.186 (0.105,0.257)**	**0.172 (0.106,0.226)**	**0.117 (0.049,0.189)**
**Number of sperm vs sperm viability**	Among individuals	-0.195 (-0.645,0.348)	-0.237 (-0.614,0.339)	0.078 (-0.416,0.601)
	Within individuals	**0.166 (0.017,0.3)**	**0.19 (0.098,0.263)**	0.149 (-0.029,0.247)

1.5.2 Supplementary Table 19: Character state model of number of sperm vs sperm viability

Testing for correlations between sperm viability and number of sperm in the effect of thermal stress using a bivariate character state model in the R-package MCMCglmm.

Type	Parameter	Posterior mode (CI)	pMCMC	Level
**Fixed effects**	traitsperm__num5	10.35 (9.8,11.01)	**0.001**	-
	traitsperm__live	-1.92 (-2.36,-1.53)	**0.001**	-
	poly(age, 2)1	8.03 (2.14,14.34)	**0.014**	-
	poly(age, 2)2	-7.27 (-12.09,-2.23)	**0.001**	-
	traitsperm__num5:TSIcatCold	-0.26 (-0.7,0.2)	0.238	-
	traitsperm__live:TSIcatCold	-0.1 (-0.37,0.17)	0.499	-
	traitsperm__num5:TSIcatHot	-0.01 (-0.44,0.46)	0.958	-
	traitsperm__live:TSIcatHot	0.03 (-0.2,0.28)	0.822	-
	traitsperm__live:poly(age, 2)1	-10.81 (-17.88,-4.14)	**0.002**	-
	traitsperm__live:poly(age, 2)2	9.2 (4.3,14.62)	**0.001**	-
**Random effect var.**	traitsperm__num5:at.level(TSIcat, Cold)	0.277 (0.14,0.749)	-	name
	traitsperm__live:at.level(TSIcat, Cold)	0.155 (0.073,0.345)	-	name
	traitsperm__num5:at.level(TSIcat, Benign)	0.311 (0.123,0.648)	-	name
	traitsperm__live:at.level(TSIcat, Benign)	0.114 (0.06,0.245)	-	name
	traitsperm__num5:at.level(TSIcat, Hot)	0.321 (0.163,0.898)	-	name
	traitsperm__live:at.level(TSIcat, Hot)	0.087 (0.046,0.213)	-	name
	traitsperm__num5	0.192 (0.086,0.983)	-	year
	traitsperm__live	0.202 (0.071,0.694)	-	year
	traitsperm__num5:at.level(TSIcat, Cold)	0.618 (0.508,0.733)	-	units
	traitsperm__live:at.level(TSIcat, Cold)	0.088 (0.065,0.107)	-	units
	traitsperm__num5:at.level(TSIcat, Benign)	0.788 (0.698,0.855)	-	units
	traitsperm__live:at.level(TSIcat, Benign)	0.032 (0.028,0.038)	-	units
	traitsperm__num5:at.level(TSIcat, Hot)	0.765 (0.631,0.875)	-	units
	traitsperm__live:at.level(TSIcat, Hot)	0.048 (0.037,0.06)	-	units
**Correlations**	traitsperm__live:at.level(TSIcat, Cold):traitsperm__num5:at.level(TSIcat, Cold).name	-0.195 (-0.645,0.348)	0.523	-
	traitsperm__live:at.level(TSIcat, Benign):traitsperm__num5:at.level(TSIcat, Benign).name	-0.237 (-0.614,0.339)	0.622	-
	traitsperm__live:at.level(TSIcat, Hot):traitsperm__num5:at.level(TSIcat, Hot).name	0.078 (-0.416,0.601)	0.681	-
	traitsperm__live:at.level(TSIcat, Cold):traitsperm__num5:at.level(TSIcat, Cold).units	0.166 (0.017,0.3)	**0.024**	-
	traitsperm__live:at.level(TSIcat, Benign):traitsperm__num5:at.level(TSIcat, Benign).units	0.19 (0.098,0.263)	**0.001**	-
	traitsperm__live:at.level(TSIcat, Hot):traitsperm__num5:at.level(TSIcat, Hot).units	0.149 (-0.029,0.247)	0.109	-

For fixed effects the posterior mean is reported

1.5.3 Supplementary Table 20: Character state model of egg-laing vs egg mass

Testing for correlations between egg mass and egg laying in the effect of thermal stress using a bivariate character state model in the R-package MCMCglmm.

Type	Parameter	Posterior mode (CI)	pMCMC	Level
**Fixed effects**	traitmass	1.44 (1.34,1.53)	**0**	-
	traitNeggs	0.17 (-0.01,0.38)	0.094	-
	dam__subpopZB	0.08 (0.05,0.11)	**0**	-
	dam__subpopCross	0.05 (0.02,0.08)	**0**	-
	TSIcatCold	-0.01 (-0.03,0)	0.109	-
	TSIcatHot	0 (-0.02,0.02)	0.929	-
	sire__subpopZB	0 (-0.01,0.01)	0.73	-
	sire__subpopCross	-0.01 (-0.03,0.01)	0.558	-
	dam__age__z	0 (-0.01,0)	0.176	-
	dam__subpopZB:TSIcatCold	0.01 (-0.04,0.06)	0.58	-
	dam__subpopCross:TSIcatCold	-0.01 (-0.05,0.03)	0.651	-
	dam__subpopZB:TSIcatHot	0 (-0.05,0.04)	0.843	-
	dam__subpopCross:TSIcatHot	0 (-0.03,0.04)	0.874	-
	traitNeggs:dam__subpopZB	-0.57 (-0.78,-0.38)	**0**	-
	traitNeggs:dam__subpopCross	-0.24 (-0.39,-0.07)	**0.007**	-
	traitNeggs:TSIcatCold	0.1 (0,0.21)	0.067	-
	traitNeggs:TSIcatHot	-0.47 (-0.56,-0.36)	**0**	-
	traitNeggs:sire__subpopZB	-0.18 (-0.3,-0.06)	**0.004**	-
	traitNeggs:sire__subpopCross	-0.21 (-0.41,-0.02)	**0.034**	-
	traitNeggs:dam__age__z	0.02 (-0.01,0.06)	0.221	-
	traitNeggs:dam__subpopZB:TSIcatCold	0.01 (-0.3,0.34)	0.944	-
	traitNeggs:dam__subpopCross:TSIcatCold	-0.29 (-0.54,-0.03)	**0.024**	-
	traitNeggs:dam__subpopZB:TSIcatHot	0.1 (-0.18,0.41)	0.491	-
	traitNeggs:dam__subpopCross:TSIcatHot	0.35 (0.1,0.59)	**0.007**	-

(continued)

Type	Parameter	Posterior mode (CI)	pMCMC	Level
**Random effect var.**	traitmass:at.level(TSIcat, Cold)	0.013 (0.011,0.015)	-	damid
	traitNeggs:at.level(TSIcat, Cold)	0.238 (0.156,0.351)	-	damid
	traitmass:at.level(TSIcat, Benign)	0.013 (0.011,0.015)	-	damid
	traitNeggs:at.level(TSIcat, Benign)	0.284 (0.227,0.356)	-	damid
	traitmass:at.level(TSIcat, Hot)	0.014 (0.013,0.017)	-	damid
	traitNeggs:at.level(TSIcat, Hot)	0.292 (0.213,0.39)	-	damid
	traitmass	0.007 (0.006,0.008)	-	enclosure
	traitNeggs	0.126 (0.097,0.171)	-	enclosure
	traitmass	0.045 (0.026,0.087)	-	year
	traitNeggs	0.151 (0.078,0.282)	-	year
	traitmass:at.level(TSIcat, Cold)	0.006 (0.006,0.007)	-	units
	traitNeggs:at.level(TSIcat, Cold)	0.832 (0.718,0.966)	-	units
	traitmass:at.level(TSIcat, Benign)	0.003 (0.003,0.003)	-	units
	traitNeggs:at.level(TSIcat, Benign)	0.511 (0.46,0.562)	-	units
	traitmass:at.level(TSIcat, Hot)	0.004 (0.004,0.005)	-	units
	traitNeggs:at.level(TSIcat, Hot)	0.72 (0.643,0.827)	-	units
**Correlations**	traitNeggs:at.level(TSIcat, Cold):traitmass:at.level(TSIcat, Cold).damid	0.068 (-0.098,0.186)	0.589	-
	traitNeggs:at.level(TSIcat, Benign):traitmass:at.level(TSIcat, Benign).damid	0.105 (-0.015,0.193)	0.089	-
	traitNeggs:at.level(TSIcat, Hot):traitmass:at.level(TSIcat, Hot).damid	0.147 (-0.011,0.251)	0.074	-
	traitNeggs:at.level(TSIcat, Cold):traitmass:at.level(TSIcat, Cold).units	0.186 (0.105,0.257)	**0**	-
	traitNeggs:at.level(TSIcat, Benign):traitmass:at.level(TSIcat, Benign).units	0.172 (0.106,0.226)	**0**	-
	traitNeggs:at.level(TSIcat, Hot):traitmass:at.level(TSIcat, Hot).units	0.117 (0.049,0.189)	**0**	-

For fixed effects the posterior mean is reported

1.5.4 Supplementary Table 21: Random regression model of number of sperm vs sperm viability

Testing for correlations between sperm viability and number of sperm in the effect of thermal stress using a bivariate random slope model in the R-package MCMCglmm.

Type	Parameter	Posterior mode (CI)	pMCMC	Level
**Fixed effects**	traitsperm__num5	10.38 (9.62,11.11)	**0.001**	-
	traitsperm__live	-1.93 (-2.61,-1.26)	**0.001**	-
	poly(age, 2)1	2.58 (-13.84,16.75)	0.76	-
	poly(age, 2)2	-6.42 (-20.73,9.61)	0.408	-
	traitsperm__live:poly(age, 2)1	-7.35 (-28.03,12.28)	0.473	-
	traitsperm__live:poly(age, 2)2	8.35 (-12.87,27.52)	0.39	-
	traitsperm__num5:TSI:StressCold	-0.5 (-1.1,0.06)	0.109	-
	traitsperm__live:TSI:StressCold	-0.2 (-0.53,0.19)	0.319	-
	traitsperm__num5:TSI:StressHeat	-0.24 (-0.85,0.41)	0.436	-
	traitsperm__live:TSI:StressHeat	0.16 (-0.22,0.55)	0.406	-
**Random effect var.**	traitsperm__num5:TSI:at.level(Stress, Heat)	0.522 (0.186,1.541)	-	name
	traitsperm__live:TSI:at.level(Stress, Heat)	0.385 (0.117,0.81)	-	name
	traitsperm__num5:TSI:at.level(Stress, Cold)	0.431 (0.155,0.894)	-	year__name
	traitsperm__live:TSI:at.level(Stress, Cold)	0.213 (0.116,0.446)	-	year__name
	traitsperm__num5:TSI:at.level(Stress, Heat)	0.73 (0.253,1.73)	-	year__name
	traitsperm__live:TSI:at.level(Stress, Heat)	0.267 (0.128,0.442)	-	year__name
	traitsperm__num5:TSI:at.level(Stress, Cold)	0.584 (0.209,1.569)	-	name
	traitsperm__live:TSI:at.level(Stress, Cold)	0.33 (0.155,0.8)	-	name
	traitsperm__num5	0.222 (0.071,1.004)	-	year
	traitsperm__live	0.152 (0.063,0.793)	-	year
	traitsperm__num5	0.691 (0.641,0.751)	-	units
	traitsperm__live	0.037 (0.032,0.041)	-	units
**Correlations**	traitsperm__live:TSI:at.level(Stress, Heat):traitsperm__num5:TSI:at.level(Stress, Heat).name	-0.071 (-0.633,0.497)	0.766	-
	traitsperm__live:TSI:at.level(Stress, Cold):traitsperm__num5:TSI:at.level(Stress, Cold).name	-0.052 (-0.603,0.526)	0.871	-
	traitsperm__live:traitsperm__num5.units	0.175 (0.095,0.224)	**0.001**	-
	traitsperm__live:TSI:at.level(Stress, Cold):traitsperm__num5:TSI:at.level(Stress, Cold).year__name	-0.04 (-0.425,0.447)	0.952	-
	traitsperm__live:TSI:at.level(Stress, Heat):traitsperm__num5:TSI:at.level(Stress, Heat).year__name	-0.08 (-0.398,0.494)	0.952	-

For fixed effects the posterior mean is reported

1.5.5 Supplementary Table 22: Random regression model of egg laying vs egg mass

Testing for correlations between egg mass and egg laying in the effect of thermal stress using a bivariate random slope model in the R-package MCMCglmm.

Type	Parameter	Posterior mode (CI)	pMCMC	Level
**Fixed effects**	traitmass	1.47 (1.35,1.59)	**0**	-
	traitNeggs	0.1 (-0.23,0.43)	0.53	-
	dam__subpopSAB	-0.04 (-0.09,0)	0.081	-
	dam__subpopZB	0.03 (-0.03,0.1)	0.32	-
	sire__subpopSAB	0.01 (-0.05,0.06)	0.798	-
	sire__subpopZB	0.02 (-0.04,0.08)	0.611	-
	dam__age__z	0 (-0.01,0.01)	0.587	-
	traitNeggs:sire__subpopSAB	0.24 (-0.04,0.52)	0.092	-
	traitNeggs:sire__subpopZB	0.01 (-0.3,0.34)	0.962	-
	traitNeggs:dam__age__z	0 (-0.05,0.05)	0.888	-
	traitmass:TSIcon:StressCold	-0.11 (-0.16,-0.05)	**0**	-
	traitNeggs:TSIcon:StressCold	-4.18 (-5.04,-3.37)	**0**	-
	traitmass:TSIcon:StressHeat	0.02 (-0.01,0.05)	0.149	-
	traitNeggs:TSIcon:StressHeat	-1.55 (-2.03,-1.13)	**0**	-
	traitmass:dam__subpopSAB:TSIcon:StressCold	0.01 (-0.05,0.07)	0.782	-
	traitNeggs:dam__subpopSAB:TSIcon:StressCold	1.49 (0.6,2.44)	**0.001**	-
	traitmass:dam__subpopZB:TSIcon:StressCold	0.04 (-0.05,0.13)	0.454	-
	traitNeggs:dam__subpopZB:TSIcon:StressCold	0.5 (-0.9,1.77)	0.463	-
	traitmass:dam__subpopSAB:TSIcon:StressHeat	-0.02 (-0.05,0.01)	0.266	-
	traitNeggs:dam__subpopSAB:TSIcon:StressHeat	-0.06 (-0.54,0.46)	0.794	-
	traitmass:dam__subpopZB:TSIcon:StressHeat	-0.06 (-0.11,-0.01)	**0.02**	-
	traitNeggs:dam__subpopZB:TSIcon:StressHeat	-1.1 (-1.82,-0.4)	**0.001**	-
**Random effect var.**	traitmass:TSIcon:at.level(Stress, Heat)	0.015 (0.014,0.018)	-	damid
	traitNeggs:TSIcon:at.level(Stress, Heat)	1.879 (1.231,2.491)	-	damid
	traitmass:TSIcon:at.level(Stress, Cold)	0.034 (0.027,0.041)	-	damid
	traitNeggs:TSIcon:at.level(Stress, Cold)	0.657 (0.237,2.356)	-	damid
	traitmass:TSIcon:at.level(Stress, Heat)	0.014 (0.013,0.015)	-	year__damid
	traitNeggs:TSIcon:at.level(Stress, Heat)	6.547 (5.83,7.352)	-	year__damid
	traitmass:TSIcon:at.level(Stress, Cold)	0.049 (0.043,0.059)	-	year__damid
	traitNeggs:TSIcon:at.level(Stress, Cold)	14.405 (11.016,18.023)	-	year__damid
	traitmass	0.009 (0.007,0.01)	-	enclosure
	traitNeggs	0.109 (0.073,0.157)	-	enclosure
	traitmass	0.045 (0.024,0.087)	-	year
	traitNeggs	0.12 (0.064,0.259)	-	year
	traitmass	0.002 (0.002,0.002)	-	units
	traitNeggs	0.211 (0.186,0.23)	-	units

(continued)

Type	Parameter	Posterior mode (CI)	pMCMC	Level
	traitNeggs:TSIcon:at.level(Stress, Heat):traitmass:TSIcon:at.level(Stress, Heat).damid	0.135 (0.031,0.269)	**0.015**	-
	traitNeggs:TSIcon:at.level(Stress, Cold):traitmass:TSIcon:at.level(Stress, Cold).damid	0.087 (-0.129,0.27)	0.515	-
**Correlations**	traitNeggs:traitmass.units	0.067 (0.025,0.104)	**0.003**	-
	traitNeggs:TSIcon:at.level(Stress, Heat):traitmass:TSIcon:at.level(Stress, Heat).year__damid	0.218 (0.128,0.288)	**0**	-
	traitNeggs:TSIcon:at.level(Stress, Cold):traitmass:TSIcon:at.level(Stress, Cold).year__damid	0.25 (0.105,0.368)	**0.001**	-

For fixed effects the posterior mean is reported

2 Supplementary Figures

2.1 Supplementary Fig. 1: Identification of critical thermal window for egg traits

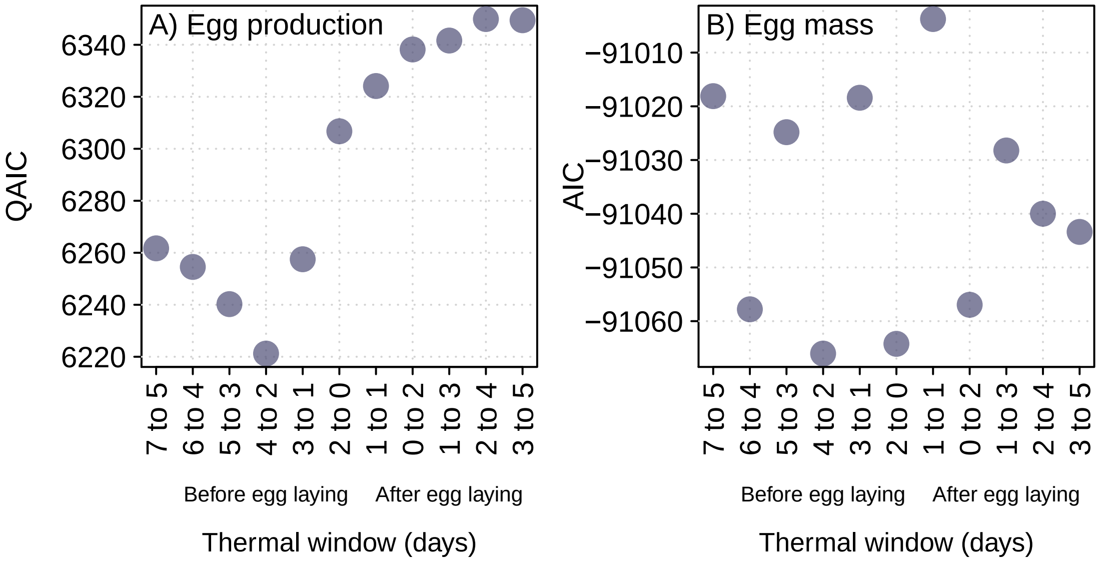

The sensitivity of female and male fertility traits to local temperature fluctuations may not only be a product of the immediate environment, but also time-lag effects. When comparing population-level GLMs using different windows to predict the sensitivity of egg production to fluctuating temperatures, we found improved predictive power of the days preceding egg-laying compared to the days around egg-laying **(A)**. AVG-T_{MAX} of the window spanning 4 to 2 days before egg-laying was the best predictor of egg-laying (i.e. the critical thermal window). This is in accordance with the duration of egg formation in the ostrich being approximately 2 days. For egg mass **(B)** the ranking of AIC was highly sensitive to outliers and we therefore removed eggs laid at the 0.5% hottest and 0.5% coldest days. The critical thermal windows of 2 to 0 days before egg-laying was choosen the best predictor, but several windows show similar performance.

2.2 Supplementary Fig. 2: Identification of critical thermal window for sperm traits

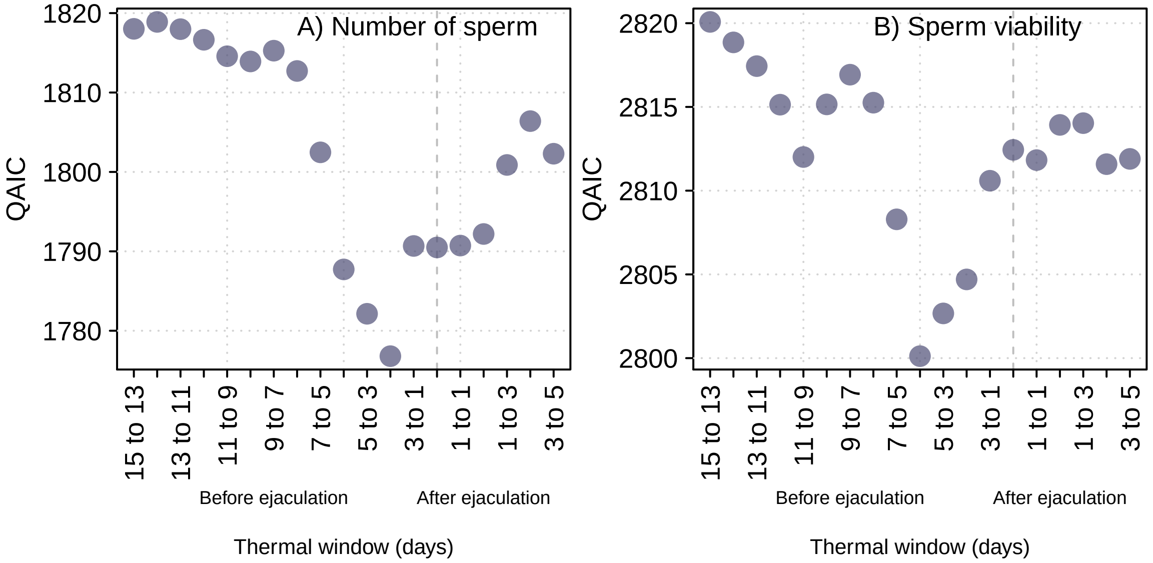

For the number of sperm **(A)** produced by males, we also found improved model fit in the days before ejaculation, with AVG-T_{MAX} of the window spanning 4 to 2 days before delivery shown to be the critical thermal window. Sperm viability **(B)** showed a similar pattern but with the best model fit using AVG-T_{MAX} being the window spanning 6 to 4 days before ejaculation. The vertical lines indicate the time of egg-laying or time of ejaculation.

2.3 Supplementary Fig. 3: The relationship between ambient temperature and egg-laying.

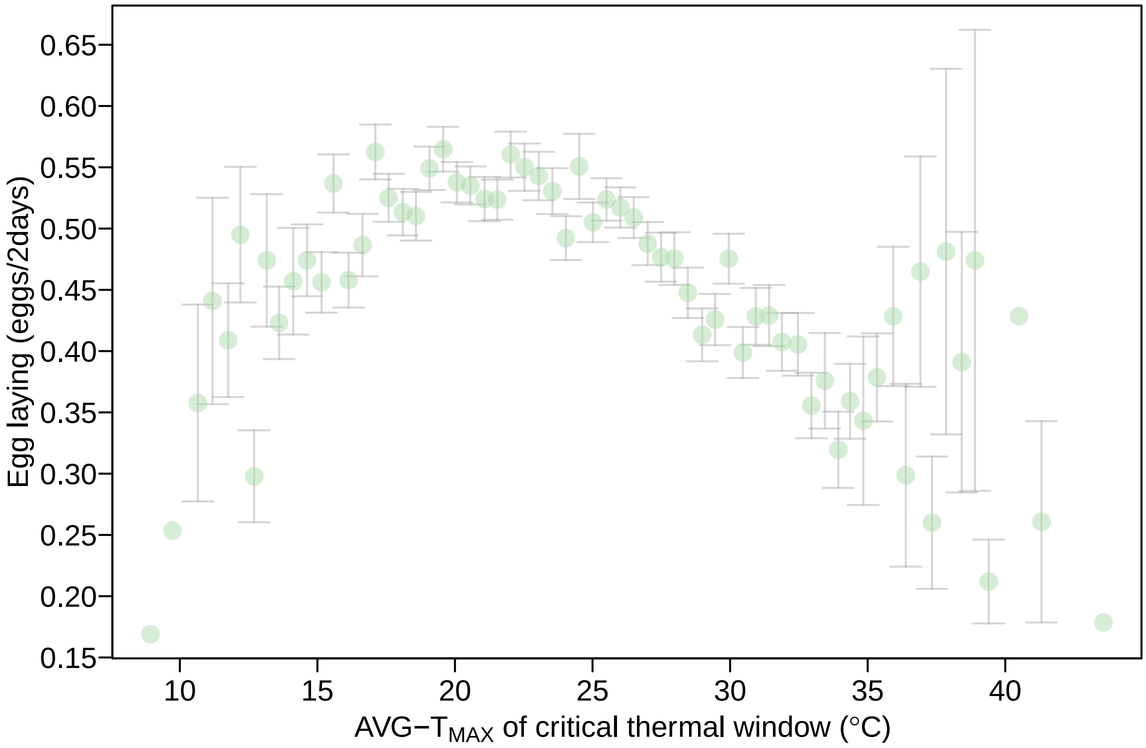

Points show observed probabilities of egg laying (n = 652 females) for the binned temperature variable. Data are presented as mean values +/- SEM.

2.4 Supplementary Fig. 4: The relationship between ambient temperature and number of sperm.

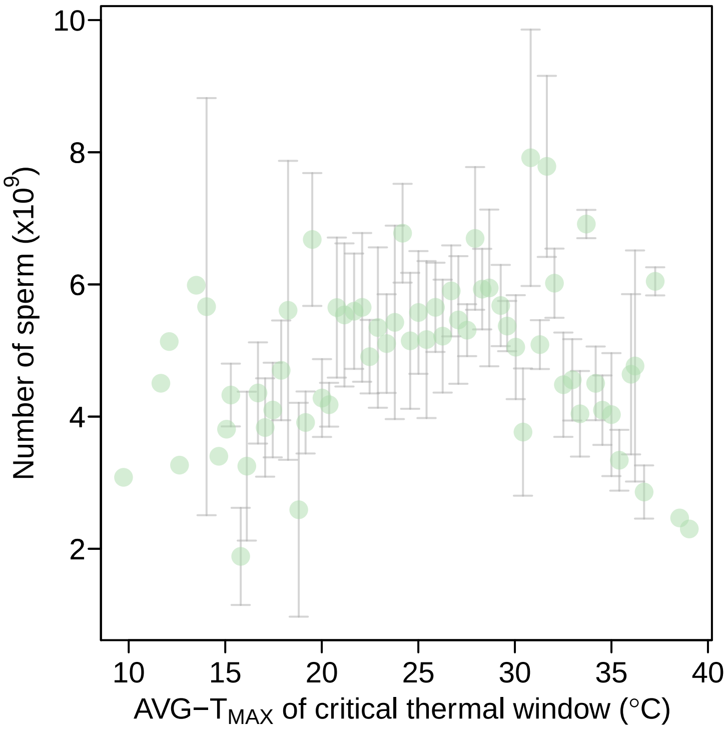

Points show observed number of sperm (n = 22 males) for the binned temperature variable. Data are presented as mean values +/- SEM.

2.5 Supplementary Fig. 5: Acclimation duration in the start of a new breeding season.

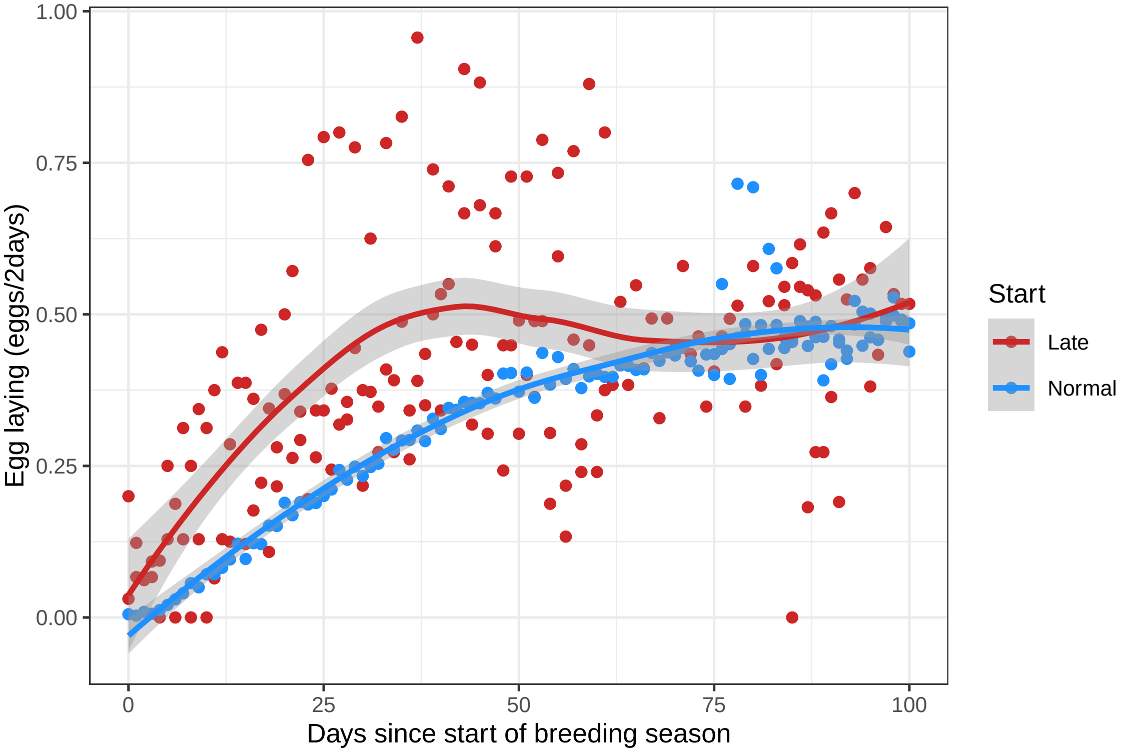

When ostrich pairs are established in the enclosures in May/June (normal start: 746 females) there is a steep increase in rate of egg-laying for the first 75 days. This increase likely reflects two effects, one being the increase in temperature at this time of year and the other being the acclimation to the enclosure and assigned partner. To determine the duration of acclimation to enclosure and partner, we investigated the reaction norm of ostrich pairs that, for various reasons, started their breeding season between July and October (Late start: 93 females) (note that these pairs are not included in the analyses presented in the main document). These late starters also show an increase in laying probability at the start and seem to be fully acclimated around 45 days after they were assigned to an enclosure. Estimated probability and 95% confidence band were obtained from a cubic spline model. Days after 100 days since start of breeding season are not shown.

2.6 Supplementary Fig. 6: Female age influences egg-laying.

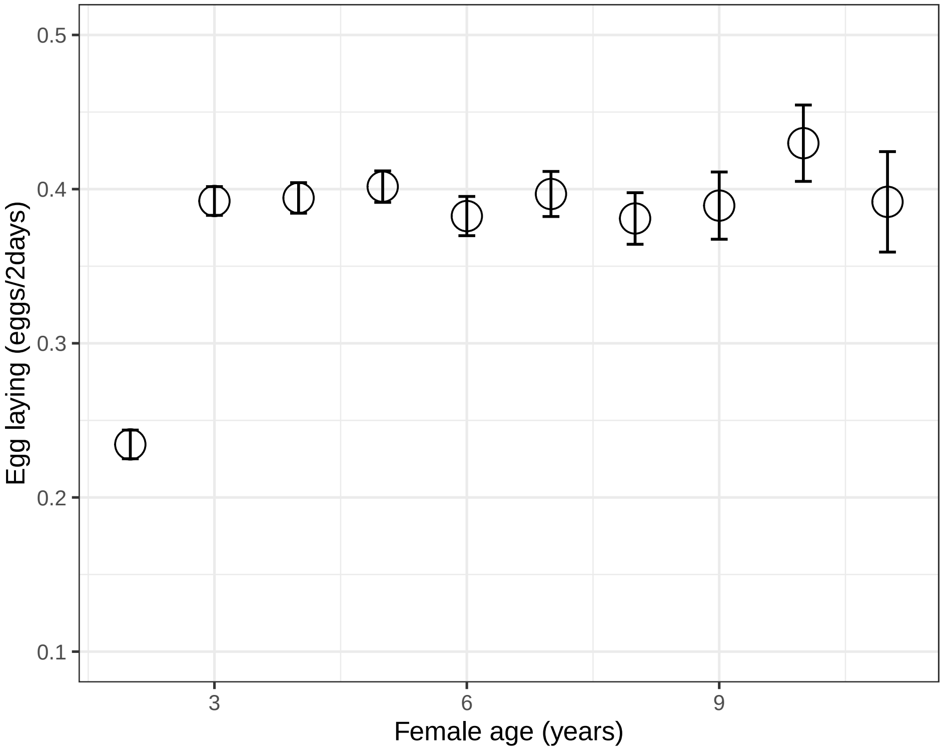

Data are presented as mean values +/- SEM across females (n = 756 females). Only 7 females had an age > 11 and are not shown.

2.7 Supplementary Fig. 7: Inspecting ranking of one-day and two-day thermal windows in egg-laying

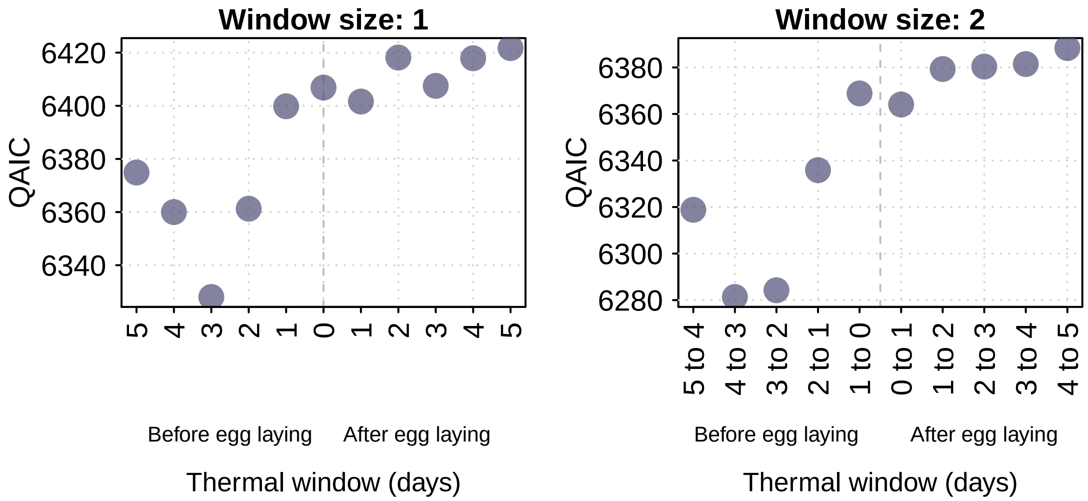

The temperature at several days before are important for egg-laying, showing that a window of three days is an acceptable way to capture the most important immediate thermal fluctuations. Best thermal windows (lowest QAIC values) are consistent with the critical three-day thermal window identified in **Supplementary Fig. 2**.

2.8 Supplementary Fig. 8: Change in egg mass with number of days since previous egg.

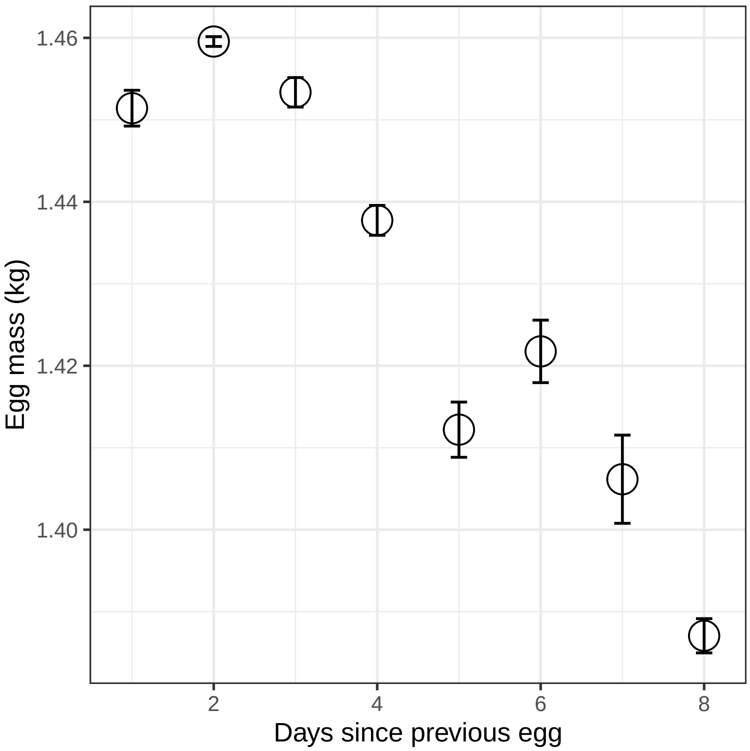

When more than three days pass since the previous egg was laid, the egg mass of the next egg starts to decline. A decrease of 70 g is substantial, since the average standard deviation of egg mass for an ostrich pair amounts to 73 g. If more than eight days passed since the previous egg, we assigned eight days since previous egg for illustration purposes. Data are presented as mean values +/- SEM across females (n = 652).
