# Supplementary material for: Extreme temperatures compromise male and female fertility in a large desert bird
Source: Nat Commun. 2021 Feb 2;12:666. doi: 10.1038/s41467-021-20937-7 (PMC7854745; doi:10.1038/s41467-021-20937-7)
Supplement: Supplementary file 3 — Description of Additional Supplementary Files [file 41467_2021_20937_MOESM3_ESM.pdf]

Description of Additional Supplementary Files

File Name: Supplementary Code 1
Description: R code used for statistical analyses
