# Supplementary material for: Extreme temperatures compromise male and female fertility in a large desert bird
Source: Nat Commun. 2021 Feb 2;12:666. doi: 10.1038/s41467-021-20937-7 (PMC7854745; doi:10.1038/s41467-021-20937-7)
Supplement: Supplementary file 5 — Reporting Summary [file 41467_2021_20937_MOESM5_ESM.pdf]

nature research

Corresponding author(s): Mads F. Schou

Last updated by author(s): Dec 11, 2020

Reporting Summary

Nature Research wishes to improve the reproducibility of the work that we publish. This form provides structure for consistency and transparency in reporting. For further information on Nature Research policies, see our Editorial Policies and the Editorial Policy Checklist.

Statistics

For all statistical analyses, confirm that the following items are present in the figure legend, table legend, main text, or Methods section.

n/a	Confirmed	
☐	☒	The exact sample size (n) for each experimental group/condition, given as a discrete number and unit of measurement
☐	☒	A statement on whether measurements were taken from distinct samples or whether the same sample was measured repeatedly
☐	☒	The statistical test(s) used AND whether they are one- or two-sided<br>*Only common tests should be described solely by name; describe more complex techniques in the Methods section.*
☐	☒	A description of all covariates tested
☐	☒	A description of any assumptions or corrections, such as tests of normality and adjustment for multiple comparisons
☐	☒	A full description of the statistical parameters including central tendency (e.g. means) or other basic estimates (e.g. regression coefficient) AND variation (e.g. standard deviation) or associated estimates of uncertainty (e.g. confidence intervals)
☐	☒	For null hypothesis testing, the test statistic (e.g. F, t, r) with confidence intervals, effect sizes, degrees of freedom and P value noted<br>*Give P values as exact values whenever suitable.*
☐	☒	For Bayesian analysis, information on the choice of priors and Markov chain Monte Carlo settings
☐	☒	For hierarchical and complex designs, identification of the appropriate level for tests and full reporting of outcomes
☐	☒	Estimates of effect sizes (e.g. Cohen's d, Pearson's r), indicating how they were calculated

Our web collection on statistics for biologists contains articles on many of the points above.

Software and code

Policy information about availability of computer code

Data collection	No software was used
Data analysis	R-packages MCMCglmm (v.2.29) and QGglmm (v. 0.7.4) were used for analyses in R (v.3.6.0). See also custom code available as a separate file.

For manuscripts utilizing custom algorithms or software that are central to the research but not yet described in published literature, software must be made available to editors and reviewers. We strongly encourage code deposition in a community repository (e.g. GitHub). See the Nature Research guidelines for submitting code & software for further information.

Data

Policy information about availability of data

All manuscripts must include a data availability statement. This statement should provide the following information, where applicable:

- Accession codes, unique identifiers, or web links for publicly available datasets
- A list of figures that have associated raw data
- A description of any restrictions on data availability

The data that support the findings of this study are available from the Western Cape Department of Agriculture in South Africa (WCDA). Restrictions apply to the use of these data, and so are not publicly available. Data are however available from the WCDA upon request.
Source data are provided within this paper for figures 2 and 3.

Field-specific reporting

Please select the one below that is the best fit for your research. If you are not sure, read the appropriate sections before making your selection.

☐ Life sciences ☐ Behavioural & social sciences ☒ Ecological, evolutionary & environmental sciences

For a reference copy of the document with all sections, see nature.com/documents/nr-reporting-summary-flat.pdf

Ecological, evolutionary & environmental sciences study design

All studies must disclose on these points even when the disclosure is negative.

Study description	We examine how temperature fluctuations over a 20-year period affect multiple fertility traits (egg number, egg mass, sperm number, sperm viability) in ostriches (n = 1299). Specifically, we tested the effect of increasing or decreasing temperatures on traits. Female fertility traits were investigated in 652 females and male fertility traits in 22 males.
Research sample	Male and female ostriches (Struthio camelus) at Oudtshoorn Research Farm, where partners and experimental conditions are controlled while individuals are exposed to natural variation in temperature. Ostriches were kept in pairs of one male and one female, or as solitary males.
Sampling strategy	We used data from all individuals present as pairs or solitary males at the Oudtshoorn Research Farm from 1998-2018 and then filtered the data as described in “Data Exclusions”. No sample-size calculation was performed.
Data collection	Data collected on ostriches was done by workers at the Oudtshoorn Research Farm. Pairs were checked twice a day and any eggs were collected and weighed using an electronic balance (Mercer). This gave us an estimate of the daily changes in quantity and mass of eggs. To control incubation effects, eggs were artificially incubated in an on-site hatchery until hatching. Eggs were stored (1-6 days) at 17ºC and 80-90% humidity with two daily rotations through a 180º angle until eggs were moved to incubators once a week. Eggs were incubated at 36.2ºC and 24% humidity with hourly rotation on their long axis through a 60º angle for the first 35 days and then switched to a hatcher set at 36ºC and 24% humidity for the remaining 7 days. Hereafter it was noted if a chick hatched successfully or not. Data collection from the solitary males was done by Maud Bonato and Pfunzo T. Muvhali. Natural ejaculates were collected from solitary males kept in individual enclosures. The number of sperm and sperm viability were measured. Semen collection was performed three to five times a week.
Timing and spatial scale	All samples are from Oudtshoorn Research Farm. Data from pairs were collected continuously from from 1998-2018. Data from males were collected continuously from 2008, when the solitary male setup was established, until 2017. In years 2012-2015 few sperm collections were completed and few of these have been analysed in the laboratory to estimate sperm viability, and this therefore represents a gap in the data collection.
Data exclusions	In two years, the laying season was extended beyond February until April. All data from these months were removed to ensure data were consistent with other years. We also removed data from pairs where the male or female was replaced during the breeding season, which occurred sometimes when individuals were injured or died. Data on the rate of egg laying from these replacement pairs indicated that acclimation to enclosure and partner takes approximately 45 days (Fig S5). Based on this information we removed data from the first 45 days from each season. Two-year old females had substantially lower reproductive success than older breeders (Fig S6, see also Cloete et al. 2006), and these were also removed from the data. Pairs that spent fewer than 200 days in their enclosure in a given year were removed so that data were consistent across pairs and years. Pairs that laid fewer than ten eggs per year were removed to avoid including incompatible pairs and individuals not in breeding condition. Semen collections were performed three to five times a week and after periods of sexual rest the first three ejaculates collected were discarded. From the resulting set of ejaculates we kept data on the first ejaculate collected each day, typically obtained in the morning, from each individual. Only males from which we were able to obtain at least five ejaculates were included in the analysis to avoid including males not accustomed to the ejaculation collection process.
Reproducibility	We cannot directly replicate the full natural experiment, where natural fluctuations in temperature is the predictor of interest. Instead the dataset consists of data from 20 years, each representing a replication of the effects of temperature on reproductive success. All 20 years were included in dataset to ensure that a random or biased pattern in one year would not affect the conclusions.<br><br>R scripts are included to facilitate reproducibility of the analyses. For each analysis, three independent MCMC chains were run to ensure results were consistent.
Randomization	There are several levels of replication within our dataset. Enclosures, each with two ostriches, represent an experimental unit. The ostriches monitored within a year can also be considered an experimental unit. Randomization took place by monitoring individual ostriches for several years and in different enclosures and with different partners. Both enclosure and year were included as random effects (intercept and slopes in unstructured variance covariance matrices) in the analysis as described in more details in the main text.
Blinding	The investigator in charge of data analysis was not involved in the data collection. There is no "treatment" in this experiment. This is a natural experiment where the differences among individuals in their response to the naturally fluctuating temperatures are quantified. Hence the need for classical "blinding" is not present.

Did the study involve field work? ☐ Yes ☒ No

Reporting for specific materials, systems and methods

We require information from authors about some types of materials, experimental systems and methods used in many studies. Here, indicate whether each material, system or method listed is relevant to your study. If you are not sure if a list item applies to your research, read the appropriate section before selecting a response.

Materials & experimental systems

n/a	Involved in the study
☒	☐ Antibodies
☒	☐ Eukaryotic cell lines
☒	☐ Palaeontology and archaeology
☐	☒ Animals and other organisms
☒	☐ Human research participants
☒	☐ Clinical data
☒	☐ Dual use research of concern

Methods

n/a	Involved in the study
☒	☐ ChIP-seq
☒	☐ Flow cytometry
☒	☐ MRI-based neuroimaging

Animals and other organisms

Policy information about studies involving animals; ARRIVE guidelines recommended for reporting animal research

Laboratory animals	Male and female ostriches (Struthio camelus) of age 2-12. Not held in a laboratory but in enclosures of Karoo shrub.
Wild animals	The study did not involve wild animals.
Field-collected samples	The study did not involve samples collected in the field.
Ethics oversight	Western Cape Department of Agriculture

Note that full information on the approval of the study protocol must also be provided in the manuscript.
